# Laboratory Confirmation of Respiratory Syncytial Virus Infection Is Not Associated With an Increased Risk of Death in Adults With Acute Respiratory Illness

**DOI:** 10.1093/ofid/ofaf004

**Published:** 2025-01-15

**Authors:** Jeffrey A Kline, Robert D Welch, Christopher Kabrhel, Daniel Mark Courtney, Carlos A Camargo, RESP-LENS site investigators, Michael J Roshon, Danielle E Turner-Lawrence, Michael A Puskarich, Ka Ming Gordon Ngai, Benton R Hunter, Joseph Bledsoe, James K d'Ettiene, Stephen S Lim, Christopher W Kabrhel, Esther J Choo, Steven M H Moore, Katherine R Buck, David M Beiser, James G Galbraith, Joby E Thoppil, Chris Kelly, Edward W Castillo, Israel E Green-Hopkins, Kristen S Nordenholz, Faheem C Guirgis, Bryan Wilson, Michael L Pulia, Stacey House, Justine M Pagenhardt

**Affiliations:** Department of Emergency Medicine, Wayne State University, Detroit, Michigan, USA; Department of Emergency Medicine, Wayne State University, Detroit, Michigan, USA; Department of Emergency Medicine, Massachusetts General Hospital, Harvard Medical School, Boston, Massachusetts, USA; Department of Emergency Medicine, University of Texas Southwestern, Dallas, Texas, USA; Department of Emergency Medicine, Massachusetts General Hospital, Boston, Massachusetts, USA

**Keywords:** COVID-19, electronic medical records, epidemiology, mortality, respiratory syncytial virus, surveillance

## Abstract

**Background:**

Limited data have described the testing patterns and outcomes of adults (≥18 years) with acute respiratory illness (ARI) in the emergency department setting.

**Methods:**

This prospective cohort study includes patients with ARI from a program sponsored by the Centers for Disease Control and Prevention entitled Respiratory Virus Laboratory Emergency Department Network Surveillance (RESP-LENS) from August 2021 until March 2024 (91 hospitals). Patients with ARIs were identified weekly by electronic surveillance for 1 or more of 130 *ICD-10* codes that defined ARI. Patients were followed for 30 days for the primary outcomes of hospitalization and mortality. Testing for RSV with nasopharyngeal swabbing followed by reverse transcription polymerase chain reaction was done as part of usual care. Risk of 30-day mortality and RSV positivity was tested in a generalized estimating equation.

**Results:**

From 1 210 394 patients with ARI, 345 185 (28.5%) adults underwent RSV testing, which was positive in 2.4%. In adults who were RSV+, the overall mortality rate was 1.9% as compared with 2.9% in adults who were RSV−. Mortality with RSV+ status increased with age ≥65 years to 3.8% (95% CI, 3.1%–4.5%). However, in the generalized estimating equation, RSV+ status was not associated with a higher rate of hospitalization (adjusted odds, 0.79; 95% CI, .75–.84) or 30-day mortality (odds, 0.62; 95% CI, .53–.74) relative to those who were RSV–. Age ≥65 years, incremental worsening of vital signs, male sex, and heart failure were independently associated with death.

**Conclusions:**

Among adults with ARI presenting to an emergency department who were tested for RSV as part of their usual care, laboratory-confirmed RSV positivity was not associated with increased risk, including hospitalization, intensive care unit requirement, or death.

Respiratory syncytial virus (RSV) can cause symptomatic respiratory infections in persons of all ages. Acute RSV infection is thought to carry substantial morbidity and even mortality, especially among older adults [1–3]. Annually, RSV infection causes hospitalization for approximately 267 of every 100 000 adults aged >65 years [4]. Prospective studies have suggested that RSV in adults may be underdetected in terms of laboratory confirmation and is associated a 7%–8% mortality rate [5]. The present analysis focuses on the burden of illness for RSV among patients presenting for unscheduled care in the US emergency department (ED) setting.

At least 120 million Americans visit an ED each year, and about 5% to 10% of these patients present with an acute respiratory illness (ARI) [6]. In recent years, particularly since 2020, many EDs have implemented the use of polymerase chain reaction (PCR)–based laboratory assays for nasopharyngeal swabs to detect acute viral infections, including RSV. Thus, the ED setting offers a national platform to survey the prevalence of viruses in patients with laboratory-proven symptomatic viral infections. Specifically, this work leverages data from the Respiratory Virus Laboratory Emergency Department Network Surveillance (RESP-LENS) network, which is sponsored by the US Centers for Disease Control and Prevention (CDC). Details of the RESP-LENS methodology have been described [7]. This network is unique inasmuch as it prospectively collects weekly data of patients with ARI, with ongoing evaluation of data, and includes laboratory confirmation of viral infection. The current analysis reports the clinical features, positive test rate, and clinical outcomes of patients of all ages tested for RSV as part of usual medical care from a prospectively collected national sample of patients in the ED with acute respiratory complaints. The primary goal, however, is to focus on adults tested for RSV in terms of the association between RSV positivity and 30-day mortality, when adjusted for multiple factors in a multivariable analysis.

## METHODS

### Study Design

This prospective cohort study was derived from weekly reported data collected by the RESP-LENS program from 19 August 2021 to 31 March 2024 without interruption. Details of the methods used by RESP-LENS are publicly available [7]. Briefly, RESP-LENS involves the collaboration of 24 investigators representing 91 hospitals and the CDC who prospectively surveil viral infections (https://www.cdc.gov/resp-lens/dashboard/). The network began in August 2021, covering all 10 regions of the US Department of Health and Human Services.

### Patient Consent Statement

The protocol was reviewed by all site institutional review boards and was deemed nonhuman subjects research (ie, surveillance), exempted from review, or approved with expedited review. As such, written informed consent was not required.

### Patient Selection and Data Extraction

Patients were identified on the basis of 1 or more of the 130 *ICD-10* diagnosis codes defining “acute respiratory illness” coded upon patient discharge from the ED to home, admission, or observation in hospital (Supplementary Table 1). These *ICD-10* codes were derived by consensus among the investigators and CDC personnel [7]. Data were collected for the prior week (Sunday–Saturday) from 91 hospitals and uploaded to a secure server each Monday. Data were collected from ARI-qualifying patients at 2 times: first, from the index visit of the week prior and then follow-up 30 days later. If the patient was discharged on the index visit and had no further encounters, then no new 30-day data were reported, although incomplete index data could be updated if needed (most commonly from patients seen on a Saturday). Patients who were treated and released on an index visit and had no other contact with the hospital’s local surveillance network, that patient was considered to be alive and to not have had any hospitalization in terms of coding the dependent variable in the multivariable models. Patients could be enrolled more than once, and unique patients with multiple visits were identified by a securely encrypted medical record number. As a surveillance network, collected data are machine verified for completeness and formatting and then reviewed weekly by 3 CDC analysts for potential anomalies (eg, unexpected changes in testing frequency). As needed, coding updates are made on a weekly basis to account for changes in test-ordering patterns (eg, use of new viral tests or updated codes).

For each qualifying patient, an SQL code retrieved 173 fields from the index visit, followed by 136 fields 30 days later. The data were extracted directly from the data warehouses of Cerner or Epic. For the medical history fields (eg, history of asthma or chronic obstructive pulmonary disease [COPD]), fields could be coded as positive or negative when the medical record specifically contained this information. However, because the electronic medical record is less likely to systematically record negatives or the absence of the condition (eg, the patient's chart has no *ICD-10* code or listing of a medical history such as asthma), the variable was treated as “assumed negative” in the generalized estimating equation (GEE). Yet, for fields where the electronic medical record is expected to always have a value (eg, age, vital signs, demographics), if a field was empty, this was encoded as missing. The equation was then repeated in a sensitivity analysis after multiple imputations for fields with >10% missing.

### Laboratory Viral Testing

The decision to perform laboratory viral testing was at the discretion of the clinical care team at each hospital or according to local policies. All sites required mandatory viral testing for all admitted patients up until approximately March 2023, but this study includes only those patients with an acute respiratory complaint as defined by the set of 130 *ICD-10* codes. All viral tests were performed on nasal or nasopharyngeal swabs with PCR or reverse transcription PCR (RT-PCR). For RSV and other viruses, each patient could be classified as positive, negative, or not tested.

### Statistical Analysis

RESP-LENS adheres to the reporting guidelines of STROBE (Strengthening the Reporting of Observational Studies in Epidemiology) and RECORD (Reporting of Studies Conducted Using Observational Routinely-Collected Data) [8, 9]. For the population of all ages, we first eliminated patients who did not undergo RSV testing; then, for patients tested, we reported RSV positivity, intensive care requirements, and mortality, stratified by 11 age groups, as customarily reported by CDC. For risk assessment, the primary focus of this report was on adults aged ≥18 years who were tested for RSV; thus, for multivariable analysis, children (age <18 years) were excluded. We compared proportions between adults who were RSV+ and RSV− using 95% CIs.

To assess risk of severe infection, we used a GEE with the dependent variables of any hospital admission (vs discharge), intensive care unit (ICU) requirement (vs discharge), or death (binomial distribution with logit link) within 30 days of the index encounter. The primary goal of the GEE was to determine the odds for an RSV+ result on RT-PCR testing of a nasopharyngeal swab done in the ED setting. We chose a marginal model approach since the main interest was population rather than individual patients. Repeated visits by patients, nested in each hospital site, were accounted for, and various correlation matrices were considered. Additionally, we sought a priori to determine the direction and magnitude of effect for other clinical variables that are readily available in the ED and were recorded in the RESP-LENS database. Prior literature has consistently suggested that older age, a history of heart failure, lung disease, and smoking increase the risk of severe disease, and these were all selected as independent variables in the GEE [10, 11]. To our knowledge, no prior literature has examined the association of ED vital signs, and given their ubiquitous availability and general predictiveness for severity in virtually all respiratory infections, we included respiratory rate, heart rate, oxygen saturation, and systolic blood pressure in the model. Last, we included race and ethnicity in the GEE.

For the multiple imputation sensitivity analysis, 10 data sets were created by the fully conditional specification method in SAS Proc MI (discriminant function for categorical data and regression method for continuous data). We chose to create 10 data sets after observing no material difference going from 5 to 10 data sets. Empirical estimates and the accompanying covariance matrices of the parameter estimates combined from each of the 10 data sets were then used to obtain the final multiple imputation–based parameter estimates with SAS Proc MIANALYZE.

The analysis was performed with SAS version 9.4 (SAS Institute), and maps were produced with ArcGIS Pro 3.1.0 software (ESRI Inc). Figures were produced with Prism version 10.0.0 for Windows (GraphPad).

## RESULTS

### Participant Characteristics

From 5 099 064 total ED visits, there were 1 210 394 (24%) encounters at the 91 participating hospitals among patients who received an ARI-qualifying *ICD-10* code. Of the 1 120 394 encounters, 536 191 (44.2%) had a laboratory test for RSV, and among these, 26 695 were RSV+ on first encounter and 9173 were RSV+ on a repeat encounter for a total of 35 571 positive test results (6.6% positive). Table 1 presents the breakdown of RSV positivity, ICU requirement, and death for these encounters. As expected, testing was heavy in children, with 47% of tests done in patients <15 years old. Among the 180 555 children aged <15 years, 27 333 (15.1%) were RSV positive, which is significantly higher than the 2.3% positive rate for patients aged ≥15 years (for the 12.8% difference: 95% CI, 12.6%–13.0%). Mortality within 30 days was exceedingly low in the RSV+ group aged <15 years: 6 of 27 333 (0.02%; 95% CI, .008%–.05%). In contrast, mortality among the RSV+ group aged ≥65 years was 125 of 3290 (3.8%; 95% CI, 3.1%–4.5%). This reinforces the decision to limit the multivariable analysis to adults.

**Table 1.** Stratification of Respiratory Syncytial Virus Testing Results and Outcomes by Age

	All	RSV+		RSV+ Discharged		RSV+ Admitted Non-ICU		RSV+ ICU Requirement		RSV+ Death	
Age, y	No.	No.	%	No.	%	No.	%	No.	%	No.	%
<1	44 863	10 684	23.8	7142	66.8	2293	21.5	1030	9.6	1	0.0
1–4	79 338	13 442	16.9	10 605	78.9	2040	15.2	773	5.8	3	0.0
5–14	56 354	3207	5.7	2732	85.2	333	10.4	574	17.9	2	0.1
15–24	40 825	826	2.0	727	88.0	62	7.5	529	64.0	0	0.0
25–34	47 197	825	1.7	721	87.4	10	1.2	532	64.5	2	0.2
35–44	42 116	815	1.9	651	79.9	108	13.3	534	65.5	1	0.1
45–54	41 674	984	2.4	695	70.6	185	18.8	549	55.8	7	0.7
55–64	56 960	1498	2.6	854	57.0	422	28.2	579	38.7	15	1.0
65–74	59 465	1244	2.1	622	50.0	410	33.0	566	45.5	28	2.3
75–84	44 805	1278	2.9	564	44.1	460	36.0	564	44.1	52	4.1
>84	22 594	768	3.4	279	36.3	323	42.1	576	75.0	45	5.9
Total	536 191	35 571	6.6	25 592	71.9	6646	18.7	7586	21.3	156	0.4

Abbreviations: ICU, intensive care unit; RSV, respiratory syncytial virus.

The majority of testing was done with multiplex RT-PCR assays that included results of influenza A and B and COVID-19. Regarding other viral positivity among patients of all ages who were RSV–, influenza A was isolated positive (ie, RSV test result was negative) in 43 909 patients, of whom 247 (0.6%) died within 30 days; influenza B was positive in 8315, of whom 9 (0.1%) died; and COVID-19 was positive in 95 758, of whom 1845 (1.9%) died. Coinfections of RSV+ (n = 35 571) with other viruses were uncommon: influenza A, 665 (1.8%); influenza B, 147 (0.4%); and COVID-19, 1556 (4.3%). Mortality for these 3 groups was low at 1 of 665, 0 of 147, and 3 of 1556, respectively. None of these 4 viruses were detected in 356 287 patients, and the 30-day mortality of these patients was 8137 of 356 287 (2.3%). If restricted to patients ≥18 years old, none of the 4 viruses were detected in 236 733 patients, and the 30-day mortality of this subgroup was 8029 of 236 733 (3.4%).

**Figure 1.**
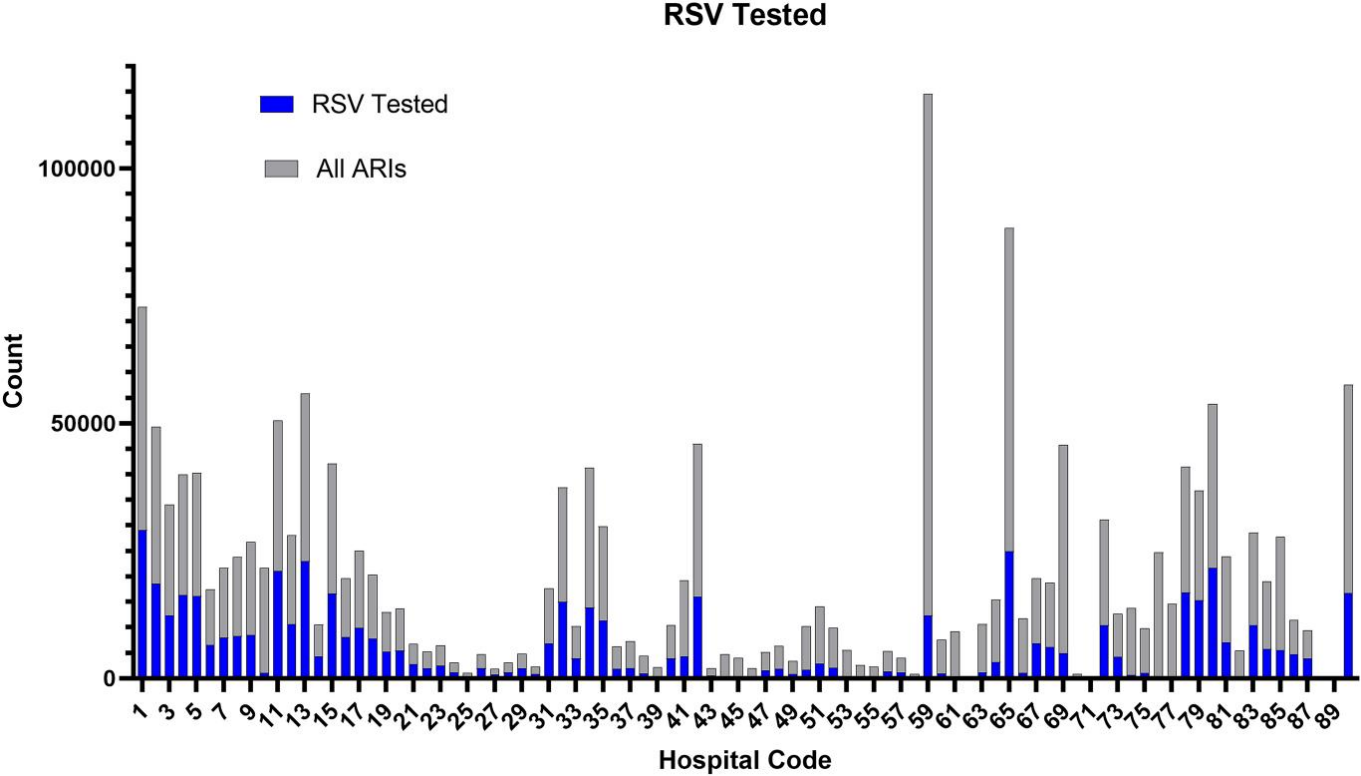
Plot showing the proportion of patients with ARI who underwent RSV testing by hospital. Abbreviations: ARI, acute respiratory illness; RSV, respiratory syncytial virus.

Mortality associated with RSV test positivity varies with the percentage of cases of ARI that are tested for RSV. Figure 1 illustrates the wide variation of RSV testing when compared among the 91 hospitals. This figure also suggests that clustering from heavy testing sites could have an outsize influence on the pooled results.

**Figure 2.**
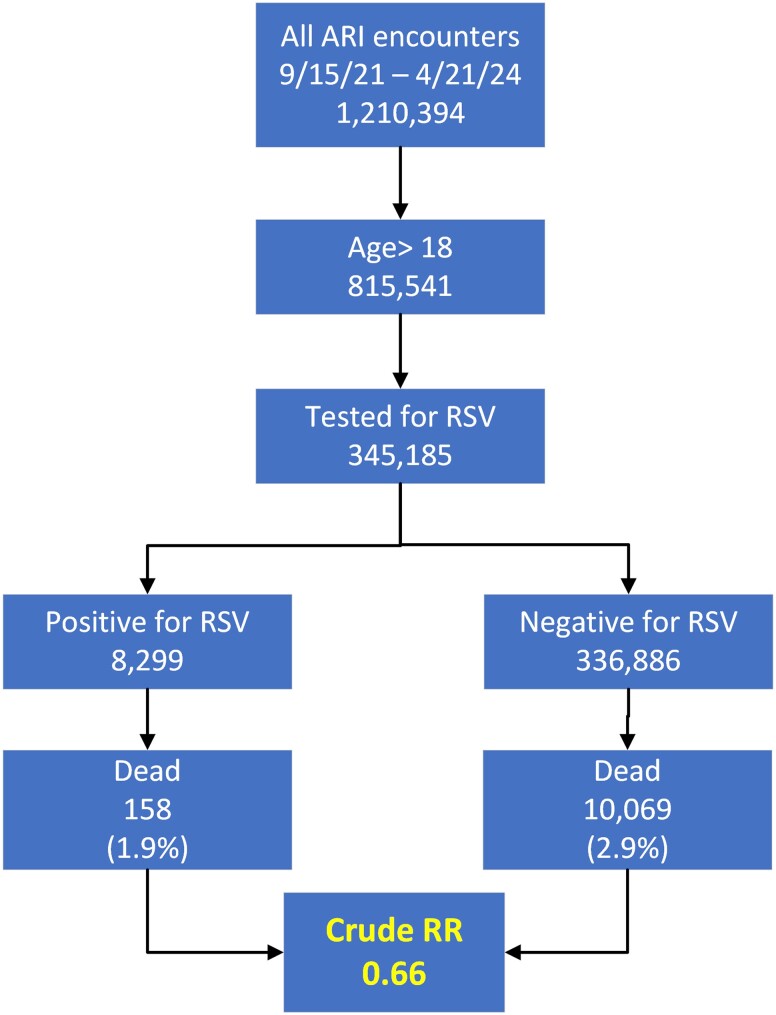
Flow diagram leading to adult population for primary analysis. Abbreviations: ARI, acute respiratory illness; RR, relative risk; RSV, respiratory syncytial virus.

The primary goal of this analysis was to determine the risk of hospital admission, ICU requirement, and death in adult patients tested for RSV to potentially allow for risk stratification at the bedside. Figure 2 shows the selection sequence of the 345 185 encounters of adults tested for RSV used in the primary analysis. An immediate observation is that the crude relative risk of death was 0.65 for the 8299 patients who were RSV+, despite being on average 4 years older than patients who were RSV–. The 345 185 encounters were made by 305 531 unique persons. Tables 2 to 4 present the vital signs, demographics, insurance status, and comorbidities for these adults, stratified by RSV status. These tables show one difference that may warrant attention from the perspective of public health. RSV positivity was significantly lower in Black adults as compared with all other races except American Indian or Alaska Native; for example, White patients had a 2.5% RSV+ rate as compared with 1.6% for Black patients (for 0.9% difference: 95% CI, .76%–1.00%)

**Table 2.** Age and Vital Signs of Adults Tested for RSV

	RSV+		RSV–	
	Mean	SD	Mean	SD
Age, y	58	20	54	21
Heart rate, beats/min	84	33	86	33
Respiratory rate, breaths/min	20.0	5.7	19.6	5.7
Oxygen saturation breathing room air	94.2	7.3	94.8	8.7
Blood pressure, mm Hg				
Systolic	141	27	135	27
Diastolic	83	18	81	19

Abbreviation: RSV, respiratory syncytial virus.

**Table 3.** Demographics and Insurance Status of Adults Tested for RSV

	RSV+		RSV−		RSV+ of Row, %^a^
Feature	No.	%	No.	%	
Sex					
Female	4128	59	166 975	50	2.5
Male	2813	41	131 543	39	2.1
Race					
American Indian or Alaska Native	43	1	2089	1	2.1
Asian	147	2	6346	2	2.3
Black	1152	17	70 799	21	1.6
Native Hawaiian or other Pacific Islander	107	2	3405	1	3.1
White	4635	67	182 124	54	2.5
More than 1 race	775	11	29 899	9	2.6
Unknown/other	77	1	2403	1	3.2
Ethnicity					
Hispanic or Latino	803	12	33 430	10	2.4
Not Hispanic or Latino	5669	82	245 393	73	2.3
Unknown	465	7	18 151	5	2.6
Insurance status					
Private or commercial	1843	27	86 008	26	2.1
Medicaid	1094	16	53 658	16	2.0
Medicare	2594	37	97 502	29	2.7
Medicaid and Medicare	44	1	2712	1	1.6
Worker's compensation	4	0	190	0	2.1
None	406	6	18 893	6	2.1
Unknown	715	10	25 650	8	2.8

Abbreviation: RSV, respiratory syncytial virus.

^a^Percentage of patients with the feature and RSV+.

**Table 4.** Comorbid Conditions of Adults Tested for RSV

	RSV+		RSV−		RSV+ of Row, %^a^
Comorbid Condition	No.	%	No.	%	
Smoker	1161	17	50 344	15	2.3
Ethanol use	528	8	26 512	8	2.0
Opioid use disorder	53	1	2209	1	2.4
Diabetes mellitus	1691	24	71 334	21	2.4
Systemic hypertension	2686	39	113 674	34	2.4
Obesity	878	13	39 428	12	2.2
Heart failure	897	13	41 385	12	2.2
Atrial fibrillation	654	9	27 360	8	2.4
Cancer	1268	18	55 260	16	2.3
Chronic obstructive pulmonary disease	808	12	35 497	11	2.3
Asthma	1013	15	41 680	12	2.4
Prior pulmonary embolism	186	3	11 167	3	1.7
Organ transplantation	144	2	5248	2	2.7
HIV or AIDS	86	1	3497	1	2.5
Death	132	2	9520	3	1.4

Abbreviation: RSV, respiratory syncytial virus.

^a^Percentage of all patients with the comorbid condition and RSV+.

The GEE model was applied to all 345 185 encounters of adult patients with ARI who underwent laboratory testing for RSV, of which 307 757 encounters remained in the final model (10.8% excluded due to missing data). When the GEE was run to assess the effect of RSV test status as an independent variable (in addition to vital signs, race, ethnicity, and prior lung disease), adult patients who were RSV+ were significantly less likely to be admitted to the hospital (odds, 0.79; 95% CI, .75–.84) and the ICU (odds 0.67; 95% CI, .60–.75). Supplementary Tables 2 and 3 show the results of these equations. Table 5 presents the results of the GEE model to assess the relative strength of the a priori–chosen factors associated with death outcome among adults tested for RSV, which resulted in an adjusted odds of 0.62 (95% CI, .53–.74). Factors in Table 5 associated with an increased risk of death included age ≥65 years, male sex, each 1-unit increase in respiratory or heart rate, each 1-unit decrease in oxygen saturation or systolic blood pressure, and history of heart failure. Factors associated with lower odds of death were non-White race and history of asthma. Regarding the association with hospital admission or ICU requirement, these same variables pointed in the same direction and with similar magnitude as in Table 5. When the GEE was rerun with death as the dependent variable and all the same independent variables in Table 5 but excluding all patients who had 1 or more confirmed infections with influenza A or B or COVID-19, the odds of RSV+ status (vs RSV–) associated with death was 0.6 (95% CI, .51–.72).

**Table 5.** Results of the Generalized Estimating Equation: Only Patients Aged ≥18 Years

	Odds Ratio for Death^a^	95% CL	
Variable		Lower	Upper
Positive RSV test result	0.62	0.53	0.74
Age, y			
≥65	3.28	3.1	3.46
18 to <65	1 [Reference]	…	…
Respiratory rate, breath/min increase			
1	1.06	1.06	1.06
5^b^	1.34	1.31	1.36
Heart rate, beat/min increase			
1	1.01	1.01	1.01
5	1.05	1.04	1.06
Oxygen saturation, % increase			
1	0.97	0.97	0.98
5	0.87	0.86	0.89
Systolic blood pressure, mm Hg increase			
1	0.98	0.98	0.98
5	0.91	0.91	0.92
Male sex	1.2	1.15	1.26
Race			
American Indian/Native Alaskan	1.17	0.87	1.58
Asian	0.8	0.67	0.95
Black/African American	0.72	0.68	0.77
Hawaiian/Pacific Islander	0.97	0.72	1.3
More than 1 race	1.03	0.79	1.34
Unknown	0.91	0.82	1.00
White	1 [Reference]	…	…
Ethnicity			
Hispanic/Latino	0.75	0.67	0.84
Unknown	0.83	0.75	0.91
Not Hispanic	1 [Reference]	…	…
Smoker	1.04	0.97	1.11
History Heart failure	1.94	1.84	2.05
Chronic obstructive pulmonary disease	0.99	0.93	1.05
Asthma	0.65	0.60	0.70

Abbreviations: CL, confidence level; RSV, respiratory syncytial virus.

^a^Odds of death.

^b^Odds of 5-unit increments are provided for more clinical relevance.

Data were missing (blank) in the following frequency: heart rate, 7.6%; respiratory rate, 0.6%; oxygen saturation, 0.4%; blood pressure, 0.5%; race, 0.5%; ethnicity, 0.5%; and age and sex, <0.1%. In the sensitivity analysis conducted after imputing missing data, the GEE results did not differ in terms of direction or significance at the 95% level for any variables. Supplementary Table 4 shows testing rates and mortality by month. As expected, RSV testing and positivity increased in winter months, but mortality rate associated with RSV+ status did not fluctuate with season.

## DISCUSSION

This analysis was conducted to provide nationally derived surveillance data to describe the epidemiology of RSV infection in US EDs. This sample provides novel data about frequency and outcomes of testing for RSV and the significance of RSV positivity on risk of 30-day mortality. From >1 million patients who presented to EDs with respiratory complaints between August 2021 and March 2024, a high proportion (44%) underwent nasopharyngeal swabbing with rapid RT-PCR testing for RSV, and of these 6.6% were positive. The data in Table 1 show the expected pattern of testing, wherein almost half of RSV testing was done in children and the positivity rate was far higher in preschool-age children than other ages. The mortality rate among children who were RSV+ was exceedingly low: 0.02% (95% CI, .008%–.05%). Table 1 reinforces the expectation that mortality rate associated with RSV+ status clearly increases with age ≥65 years [12–14]. In view of these findings and in consideration of the relative dearth of published prospective data of RSV in adults who present for unscheduled care for ARI, as well as the uncertainty surrounding RSV vaccination across adulthood, we focused the risk analysis on adults [4, 15].

The GEE model demonstrated clear evidence that in this population, RSV positivity was not associated with increased rates of hospitalization, ICU requirement, or 30-day mortality before and after adjusting for age, vital signs, sex, race, ethnicity, and comorbid conditions. Notably, the odds of mortality for adults who were RSV+ was 0.62 (95% CI, .53–.74), and this finding was unchanged after imputation of missing values. The odds did not change when RSV− cases with influenza A, influenza B, or COVID-19 infection were excluded from the GEE. The mortality rate observed in this patient sample is lower than that in several recently published reports of RSV in adults [16–18]. One difference is that prior studies reported exclusively on hospitalized older adults with a mean age consistently over 70 years, whereas only 28% of our sample was admitted to the hospital and the mean age was 58 years, when children aged <18 years were excluded. Among patients older than 65 years, the mortality rate in the present sample was 3.8% (95% CI, 3.1%–4.5%). Data in Tables 2 to 5 do not show any clear difference in testing patterns that clearly indicate that healthier patients with respiratory illness are selected for RSV testing. In a survey of site leaders, it is clear that in all 91 EDs, the common practice is to not test patients who are unlikely to require admission, thus skewing the testing toward patients who tend to have worsened illness severity. The possibility that positive test results reflect chronic carriage rather than acute infection seems unlikely. The majority of prior literature that employed serologic follow-up would argue that the main shortcoming of nasopharyngeal swabbing with RT-PCR detection is low diagnostic sensitivity, and it is unlikely that low sensitivity would impart a selection bias toward lower severity [4, 19].

We expected to find that age and vital signs would be significantly associated with death among patients tested for RSV and, as expected, they were. The importance of this is that, first, to our knowledge, no data have been forwarded to quantify the significance of vital signs for potential prediction of death in association with RSV positivity and, second, vital signs are ubiquitously available in emergency care. Accordingly, vital signs are ideal source variables for the construction of a prognostic rule for patients with RSV+ status. We did not report the results of the GEE isolated to adults who were RSV+ because we think that it is first important to understand the risks of the patients tested, given that nasopharyngeal swabbing followed by RT-PCR may miss 25% to 50% of acute RSV infections [4, 19]. From prior literature, we expected to find heart failure being strongly associated with death—and it was. Yet, the GEE did not find COPD to be significantly associated with an increased risk of death. The finding of asthma being associated with an odds ratio with a top-limit 95% CI below unity was unexpected. We do not have a firm hypothesis for the COPD and asthma findings, but we speculate that this may reflect a lower clinical threshold at which clinicians decide to test for RSV in patients with a history with COPD or asthma. To state this assertion explicitly, patients in the ED with typical exacerbations of asthma and COPD would qualify as having ARI for this study (ie, meet 1 or more of the 130 *ICD-10* codes), but they may not have typical symptoms expected for RSV. However, caution and normative behavior—that is, recognizing that RSV testing is low value but ordering anyway based on the belief that others would want the test done—may compel clinicians to widely order RSV testing in this subset [20]. We also found that male sex increased the odds of death, consistent with many prior studies demonstrating a female survival advantage in community-acquired pneumonia [21].

The findings that non-White race and Hispanic or Latino ethnicity were associated with a lower risk of death appear to be novel findings. Previous literature has reported on differences in frequency and severity of RSV infection in children of color or Latino ethnicity, but to our knowledge, ours are the first data to suggest a lower odds of death in non-White adults with RSV infection [22–24]. This finding is unexplained by any obvious differences in age, comorbid conditions, or vital signs.

The present analysis has limitations. First, the decision to test was in the hands of local clinicians, and as suggested in Figure 1, hospitals varied considerably in local thresholds to test for RSV. This may have introduced subtle biases that we could not address with our sensitivity analyses. Second, there are inherent limitations associated with using data from the electronic medical record rather than patient interview. Third, the study lacked serologic testing, which would likely have increased the rate of identification of acute RSV infection.

## CONCLUSIONS

In this large national sample of adults (age ≥18 years) presenting to the ED with ARI from August 2021 until March 2024 who were selected for testing for RSV as part of their usual care, those who had RSV-positive test results had significantly lower odds of death within 30 days as compared with patients who had RSV-negative test results.

## Supplementary Material

ofaf004_Supplementary_Data

## Supplementary Data

Supplementary materials are available at *Open Forum Infectious Diseases* online. Consisting of data provided by the authors to benefit the reader, the posted materials are not copyedited and are the sole responsibility of the authors, so questions or comments should be addressed to the corresponding author.

## Notes

***Acknowledgments.*** The following principal investigators were involved in the RESP-LENS effort (to be accessible as “collaborators” in PubMed) and are listed here alphabetically by institution: Michael J. Roshon, Centura Health; Danielle E. Turner-Lawrence, Corewell Health William Beaumont University Hospital; Michael A. Puskarich, Hennepin Healthcare; Ka Ming Gordon Ngai, Icahn School of Medicine at Mount Sinai; Benton R. Hunter, Indiana University School of Medicine; Joseph Bledsoe, Intermountain Healthcare; James K. d’Ettiene, JPS Health Network; Stephen S. Lim, LCMC Health; Christopher W. Kabrhel, Massachusetts General Hospital; Esther J. Choo, Oregon Health & Science University; Steven MH Moore, Penn State Health; Katherine R. Buck, The Ohio State University Wexner Medical Center; David M. Beiser, The University of Chicago Department of Medicine; James G. Galbraith, The University of Mississippi Medical Center; Joby E. Thoppil, The University of Texas, Southwestern; Chris Kelly, The University of Utah; Edward W. Castillo, University of California, San Diego; Israel E. Green-Hopkins, University of California, San Francisco; Kristen S. Nordenholz, University of Colorado Anschutz Medical Campus; Faheem C. Guirgis, University of Florida Health; Bryan Wilson, University of Iowa; Michael L. Pulia, University of Wisconsin–Madison; Stacey House, Washington University School of Medicine in St Louis; Justine M. Pagenhardt, West Virginia University.

***Author contributions.*** J. A. K. conceived the work, obtained funding, organized the network, and wrote the first draft of the manuscript. R. D. W. performed the statistical analysis and edited/approved this manuscript. C. K. and D. M. C. assisted in weekly data transmission, participated in regular network meetings, and edited this manuscript. C. A. C. co-conceived the work, assisted with funding, provided technical advice for the conduct of the network and database content, and edited the manuscript.

***Data availability.*** The deidentified data set is available upon request to the corresponding author.

***Data sharing.*** The RESP-LENS data management and data-sharing plans are publicly available at https://dmptool.org/plans/70148.

***Financial support.*** This work was supported by a contract from the Centers for Disease Control and Prevention to Wayne State University (75D30121C11813; Respiratory Virus Laboratory Emergency Department Network Surveillance).
