# Supplementary material for: Laboratory Confirmation of Respiratory Syncytial Virus Infection Is Not Associated With an Increased Risk of Death in Adults With Acute Respiratory Illness
Source: Open Forum Infect Dis. 2025 Jan 15;12(2):ofaf004. doi: 10.1093/ofid/ofaf004 (PMC11800477; doi:10.1093/ofid/ofaf004)
Supplement: ofaf004_Supplementary_Data [file ofaf004_supplementary_data.zip › Supplemental Table 1-ICD codes.docx]

Supplemental Table 1. International classification of diseases 10 identifiers used to locate patients with acute respiratory infection.

| **ICD 10 Code** | **ICD 10 Description** |
| --- | --- |
| A22.1 | Pulmonary anthrax |
| A37.00 | Whooping cough due to Bordetella Pertussis without Pneumonia |
| A37.01 | Whooping cough: Bordetella pertussis, pneumonia |
| A37.10 | Whooping cough due to Bordetella parapertussis without pneumonia |
| A37.11 | Whooping cough: B. parapertussis, pneumonia |
| A37.80 | Whooping cough due to other Bordetella species without pneumonia |
| A37.81 | Whooping cough: other Bordetella, pneumonia |
| A37.90 | Whooping cough, unspecified species without pneumonia |
| A37.91 | Whooping cough, unspecified species, pneumonia |
| A48.1 | Legionnaires’ disease |
| B25.0 | Cytomegaloviral pneumonitis |
| B34.2 | Coronavirus infection, unspecified |
| B34.9 | Viral infection, unspecified |
| B44.0 | Invasive pulmonary aspergillosis |
| B44.81 | Allergic bronchopulmonary aspergillosis |
| B44.9 | Aspergillosis, unspecified |
| B97.2 | Coronavirus as the cause of diseases classified elsewhere |
| B97.4 | Respiratory syncytial virus as the cause of diseases classified elsewhere |
| J00 | Acute nasopharyngitis (common cold) |
| J01.00 | Acute maxillary sinusitis, unspecified |
| J01.01 | Acute recurrent maxillary sinusitis |
| J01.10 | Acute frontal sinusitis, unspecified |
| J01.11 | Acute recurrent frontal sinusitis |
| J01.20 | Acute ethmoidal sinusitis, unspecified |
| J01.21 | Acute recurrent ethmoidal sinusitis |
| J01.30 | Acute sphenoidal sinusitis, unspecified |
| J01.31 | Acute recurrent sphenoidal sinusitis |
| J01.40 | Acute pansinusitis, unspecified |
| J01.41 | Acute recurrent pansinusitis |
| J01.80 | Other acute sinusitis |
| J01.81 | Other acute recurrent sinusitis |
| J01.90 | Acute sinusitis, unspecified |
| J01.91 | Acute recurrent sinusitis, unspecified |
| J02.0 | Streptococcal pharyngitis |
| J02.8 | Acute pharyngitis due to other specified organisms |
| J02.9 | Acute pharyngitis, unspecified |
| J03.00 | Acute streptococcal tonsillitis, unspecified |
| J03.01 | Acute recurrent streptococcal tonsillitis |
| J03.80 | Acute tonsillitis due to other specified organisms |
| J03.81 | Acute recurrent tonsillitis due to other specified organisms |
| J03.90 | Acute tonsillitis, unspecified |
| J03.91 | Acute recurrent tonsillitis, unspecified |
| J04.0 | Acute laryngitis |
| J04.10 | Acute tracheitis without obstruction |
| J04.11 | Acute tracheitis with obstruction |
| J04.2 | Acute laryngotracheitis |
| J04.30 | Supraglottitis, unspecified, without obstruction |
| J04.31 | Supraglottitis, unspecified, with obstruction |
| J05.0 | Acute obstructive laryngitis [croup] |
| J05.10 | Acute epiglottitis without obstruction |
| J05.11 | Acute epiglottitis with obstruction |
| J06.0 | Acute laryngopharyngitis |
| J06.9 | Acute upper respiratory infection, unspecified |
| J09.X | Influenza due to identified novel influenza A virus - all sub codes |
| J10.X | Influenza due to other identified influenza virus - all sub codes |
| J11.X | Influenza due to unidentified influenza virus - all sub codes |
| J12.0 | Adenoviral pneumonia |
| J12.1 | Respiratory syncytial virus pneumonia |
| J12.2 | Parainfluenza virus pneumonia |
| J12.3 | Human metapneumovirus pneumonia |
| J12.81 | Pneumonia due to SARS-associated coronavirus |
| J12.82 | Pneumonia due to COVID-19 / SARS CoV-2 |
| J12.89 | Other viral pneumonia |
| J12.9 | Viral pneumonia, unspecified |
| J13 | Pneumonia due to Streptococcus pneumoniae |
| J14 | Pneumonia due to Hemophilus influenzae |
| J15.0 | Pneumonia due to Klebsiella pneumoniae |
| J15.1 | Pneumonia due to Pseudomonas |
| J15.20 | Pneumonia due to staphylococcus, unspecified |
| J15.211 | Pneumonia due to Methicillin susceptible Staphylococcus aureus |
| J15.212 | Pneumonia due to Methicillin resistant Staphylococcus aureus |
| J15.29 | Pneumonia due to other staphylococcus |
| J15.3 | Pneumonia due to streptococcus, group B |
| J15.4 | Pneumonia due to other streptococci |
| J15.5 | Pneumonia due to Escherichia coli |
| J15.6 | Pneumonia due to other Gram-negative bacteria |
| J15.7 | Pneumonia due to Mycoplasma pneumoniae |
| J15.8 | Pneumonia due to other specified bacteria |
| J15.9 | Unspecified bacterial pneumonia |
| J16.0 | Chlamydial pneumonia |
| J16.8 | Pneumonia due to other specified infectious organisms |
| J17 | Pneumonia in diseases classified elsewhere |
| J18.0 | Bronchopneumonia, unspecified organism |
| J18.1 | Lobar pneumonia, unspecified organism |
| J18.2 | Hypostatic pneumonia, unspecified organism |
| J18.8 | Other pneumonia, unspecified organism |
| J18.9 | Pneumonia, unspecified organism |
| J20.0 | Acute bronchitis due to Mycoplasma pneumoniae |
| J20.1 | Acute bronchitis due to Hemophilus influenzae |
| J20.2 | Acute bronchitis due to streptococcus |
| J20.3 | Acute bronchitis due to coxsackievirus |
| J20.4 | Acute bronchitis due to parainfluenza virus |
| J20.5 | Acute bronchitis due to respiratory syncytial virus |
| J20.6 | Acute bronchitis due to rhinovirus |
| J20.7 | Acute bronchitis due to echovirus |
| J20.8 | Acute bronchitis due to other specified organisms |
| J20.9 | Acute bronchitis, unspecified |
| J21.0 | Acute bronchiolitis due to respiratory syncytial virus |
| J21.1 | Acute bronchiolitis due to human metapneumovirus |
| J21.8 | Acute bronchiolitis due to other specified organisms |
| J21.9 | Acute bronchiolitis, unspecified |
| J22 | Unspecified acute lower respiratory tract infection |
| J39.8 | Other specified diseases of upper respiratory tract |
| J40 | Bronchitis, not specified as acute or chronic |
| J47.9 | Bronchiectasis with acute exacerbation |
| J80 | Acute Respiratory Distress Syndrome |
| J85.1 | Abscess of lung with pneumonia |
| J95.821 | Acute postprocedural respiratory failure |
| J96.0 | Acute respiratory failure |
| J96.00 | Acute respiratory failure, unspecified whether with hypoxia or hypercapnia |
| J96.01 | Acute respiratory failure with hypoxia |
| J96.02 | Acute respiratory failure with hypercapnia |
| J96.2 | Acute and chronic respiratory failure |
| J96.20 | Acute and chronic respiratory failure, unspecified whether with hypoxia or hypercapnia |
| J96.21 | Acute and chronic respiratory failure with hypoxia |
| J96.22 | Acute and chronic respiratory failure with hypercapnia |
| J96.91 | Respiratory failure, unspecified with hypoxia |
| J98.8 | Other specified respiratory disorders |
| R05 | Cough |
| R06.00 | Dyspnea, unspecified plus fever |
| R06.02 | Shortness of breath |
| R06.03 | Acute respiratory distress |
| R09.02 | Hypoxemia |
| R09.2 | Respiratory arrest |
| R43.0 | Anosmia |
| R43.1 | Parosmia |
| R43.2 | Parageusia |
| R50.9 | Fever, unspecified |
| U07.1 | COVID-19, virus identified |
| U07.2 | COVID-19, virus not identified |
