# Supplementary material for: Laboratory Confirmation of Respiratory Syncytial Virus Infection Is Not Associated With an Increased Risk of Death in Adults With Acute Respiratory Illness
Source: Open Forum Infect Dis. 2025 Jan 15;12(2):ofaf004. doi: 10.1093/ofid/ofaf004 (PMC11800477; doi:10.1093/ofid/ofaf004)
Supplement: ofaf004_Supplementary_Data [file ofaf004_supplementary_data.zip › Supplemental Table 3-ICU admission.docx]

00:25 Thursday, December 5, 2024 **1**

Supplemental Table 3: Odds ratios (ORs) from a generalized estimating equation with intensive care admission as the dependent variable

| **Obs** | **Parameter** | **level1** | **OR** | **LCI** | **UCI** | **Pr** **>** **\|Z\|** | **OddsRatio**  **for** **Increase** **of** **5** **Units** | **95%**  **LCL** **for** **Increase**  **of** **5** **Units** | **95%**  **UCL** **for** **Increase**  **of** **5** **Units** |
| --- | --- | --- | --- | --- | --- | --- | --- | --- | --- |
| **1** | Intercept |  | 10.49 | 7.21 | 15.26 | <.0001 | . | . | . |
| **2** | All_RSV_Test | Pos | 0.62 | 0.55 | 0.71 | <.0001 | . | . | . |
| **3** | All_RSV_Test | Neg | . | . | . | . | . | . | . |
| **4** | Cat4Age | >= 65 | 2.29 | 2.20 | 2.38 | <.0001 | . | . | . |
| **5** | Cat4Age | 18 to < 65 | . | . | . | . | . | . | . |
| **6** | rrFix |  | 1.11 | 1.11 | 1.12 | <.0001 | 1.71 | 1.68 | 1.75 |
| **7** | hrFix |  | 1.02 | 1.02 | 1.02 | <.0001 | 1.11 | 1.11 | 1.12 |
| **8** | o2Fix |  | 0.92 | 0.92 | 0.93 | <.0001 | 0.67 | 0.66 | 0.68 |
| **9** | sbpFix |  | 0.99 | 0.99 | 0.99 | <.0001 | 0.94 | 0.93 | 0.94 |
| **10** | sex | Male | 1.45 | 1.40 | 1.51 | <.0001 | . | . | . |
| **11** | sex | Female | . | . | . | . | . | . | . |
| **12** | race | American Indian/Native Alaskan | 1.26 | 0.97 | 1.63 | 0.0853 | . | . | . |
| **13** | race | Asian | 0.75 | 0.67 | 0.85 | <.0001 | . | . | . |
| **14** | race | Black/AA | 1.03 | 0.99 | 1.08 | 0.1903 | . | . | . |
| **15** | race | Hawaiian/Pacific Islander | 0.69 | 0.56 | 0.84 | 0.0003 | . | . | . |
| **16** | race | More Than One Race | 1.22 | 1.01 | 1.49 | 0.0433 | . | . | . |
| **17** | race | Unknown | 0.78 | 0.73 | 0.84 | <.0001 | . | . | . |
| **18** | race | White | . | . | . | . | . | . | . |
| **19** | ethnicity | Hispanic/Latino | 0.72 | 0.67 | 0.77 | <.0001 | . | . | . |
| **20** | ethnicity | Unknown | 1.17 | 1.09 | 1.26 | <.0001 | . | . | . |
| **21** | ethnicity | Not Hispanic | . | . | . | . | . | . | . |
| **22** | smoker | Pos | 1.15 | 1.10 | 1.21 | <.0001 | . | . | . |
| **23** | smoker | Neg | . | . | . | . | . | . | . |
| **24** | heart_failure | Pos | 4.03 | 3.84 | 4.23 | <.0001 | . | . | . |
| **25** | heart_failure | Neg | . | . | . | . | . | . | . |
| **26** | copd | Pos | 1.78 | 1.68 | 1.88 | <.0001 | . | . | . |
| **27** | copd | Neg | . | . | . | . | . | . | . |
| **28** | asthma | Pos | 0.69 | 0.65 | 0.73 | <.0001 | . | . | . |
| **29** | asthma | Neg | . | . | . | . | . | . | . |
