# Supplementary material for: Laboratory Confirmation of Respiratory Syncytial Virus Infection Is Not Associated With an Increased Risk of Death in Adults With Acute Respiratory Illness
Source: Open Forum Infect Dis. 2025 Jan 15;12(2):ofaf004. doi: 10.1093/ofid/ofaf004 (PMC11800477; doi:10.1093/ofid/ofaf004)
Supplement: ofaf004_Supplementary_Data [file ofaf004_supplementary_data.zip › Supplemental Table 4 DeathByMonthByRSV.docx]

| **Table S4: Death by Month for adults tested for RSV** | | | | | | | | | | | | | |  |
| --- | --- | --- | --- | --- | --- | --- | --- | --- | --- | --- | --- | --- | --- | --- |
|  |  |  |  |  |  |  |  |  |  |  |  |  |  |  |
| **Death** | **Month** | | | | | | | | | | | | |  |
|  |  |  |  |  |  |  |  |  |  |  |  |  |  |  |
|  | **1** | **2** | **3** | **4** | **5** | **6** | **7** | **8** | **9** | **10** | **11** | **12** | **Total** |  |
|  |  |  |  |  |  |  |  |  |  |  |  |  |  |  |
|  |  |  |  |  |  |  |  |  |  |  |  |  |  |  |
| **Yes** | 1678 | 1163 | 684 | 610 | 621 | 544 | 495 | 507 | 560 | 635 | 1052 | 1520 | 10069 |  |
| % of total | 0.5 | 0.35 | 0.2 | 0.18 | 0.18 | 0.16 | 0.15 | 0.15 | 0.17 | 0.19 | 0.31 | 0.45 | 2.99 |  |
| % of month | 3.3 | 3.33 | 2.01 | 3.17 | 3.31 | 3.28 | 3.51 | 3.41 | 3.16 | 2.99 | 2.81 | 2.66 |  |  |
| **No** | 49198 | 33786 | 33371 | 18613 | 18117 | 16052 | 13592 | 14370 | 17187 | 20616 | 36337 | 55579 | 326818 |  |
| % of total | 14.6 | 10.03 | 9.91 | 5.52 | 5.38 | 4.76 | 4.03 | 4.27 | 5.1 | 6.12 | 10.79 | 16.5 | 97.01 |  |
| % of month | 96.7 | 96.67 | 97.99 | 96.83 | 96.69 | 96.72 | 96.49 | 96.59 | 96.84 | 97.01 | 97.19 | 97.34 |  |  |
| **Total** | 50876 | 34949 | 34055 | 19223 | 18738 | 16596 | 14087 | 14877 | 17747 | 21251 | 37389 | 57099 | 336887 |  |
|  | 15.1 | 10.37 | 10.11 | 5.71 | 5.56 | 4.93 | 4.18 | 4.42 | 5.27 | 6.31 | 11.1 | 16.95 | 100 |  |

**The SAS System**

**The FREQ Procedure All_RSV_Test=Yes**

| **Frequency** | **Table of death by Month** | | | | | | | | | | | | | |
| --- | --- | --- | --- | --- | --- | --- | --- | --- | --- | --- | --- | --- | --- | --- |
| **Percent** |  |  |  |  |  |  |  |  |  |  |  |  |  |  |
|  |  | **Month** | | | | | | | | | | | | |
| **Col Pct** | **death** |  |  |  |  |  |  |  |  |  |  |  |  |  |
|  |  | **1** | **2** | **3** | **4** | **5** | **6** | **7** | **8** | **9** | **10** | **11** | **12** | **Total** |
|  | **(death)** |  |  |  |  |  |  |  |  |  |  |  |  |  |
|  |  |  |  |  |  |  |  |  |  |  |  |  |  |  |
|  | **Yes** | 42 | 10 | 0 | 4 | 0 | 0 | 0 | 0 | 4 | 7 | 25 | 66 | 158 |
|  |  | 0.51 | 0.12 | 0.00 | 0.05 | 0.00 | 0.00 | 0.00 | 0.00 | 0.05 | 0.08 | 0.30 | 0.80 | 1.90 |
|  |  | 2.64 | 1.68 | 0.00 | 5.19 | 0.00 | 0.00 | 0.00 | 0.00 | 2.99 | 1.32 | 1.24 | 2.36 |  |
|  | **No** | 1549 | 585 | 348 | 73 | 54 | 51 | 37 | 64 | 130 | 522 | 1998 | 2729 | 8140 |
|  |  | 18.67 | 7.05 | 4.19 | 0.88 | 0.65 | 0.61 | 0.45 | 0.77 | 1.57 | 6.29 | 24.08 | 32.89 | 98.10 |
|  |  | 97.36 | 98.32 | 100.00 | 94.81 | 100.00 | 100.00 | 100.00 | 100.00 | 97.01 | 98.68 | 98.76 | 97.64 |  |
|  | **Total** | 1591 | 595 | 348 | 77 | 54 | 51 | 37 | 64 | 134 | 529 | 2023 | 2795 | 8298 |
|  |  | 19.17 | 7.17 | 4.19 | 0.93 | 0.65 | 0.61 | 0.45 | 0.77 | 1.61 | 6.38 | 24.38 | 33.68 | 100.00 |
